# Temporal evolution of hemodynamics in murine arteriovenous fistula: A micro-CT based CFD study

**DOI:** 10.1371/journal.pcbi.1012985

**Published:** 2025-08-18

**Authors:** Lianxia Li, Unimunkh Uriyanghai, Christine Wai, Hong Yuan, Eric W. Livingston, Edward M. Bahnson, Vinay Sudarsanam, Samuel Haddad, Prabir Roy-Chaudhury, Boyce E. Griffith, Gang Xi

**Affiliations:** 1 UNC Kidney Center, University of North Carolina, Chapel Hill, North Carolina, United States of America; 2 Carolina Center for Interdisciplinary Applied Mathematics, University of North Carolina, Chapel Hill, North Carolina, United States of America; 3 Department of Radiology, University of North Carolina, Chapel Hill, North Carolina, United States of America; 4 Biomedical Research Imaging Center, University of North Carolina, Chapel Hill, North Carolina, United States of America; 5 Department of Cell Biology and Physiology. University of North Carolina, Chapel Hill, North Carolina, United States of America; 6 McAllister Heart Institute, University of North Carolina School of Medicine, Chapel Hill, North Carolina, United States of America; 7 WG (Bill) Hefner VA Medical Center, Salisbury, North Carolina, United States of America; 8 Department of Mathematics, University of North Carolina, Chapel Hill, North Carolina, United States of America; 9 Department of Biomedical Engineering, University of North Carolina, Chapel Hill, North Carolina, United States of America; 10 Computational Medicine Program, University of North Carolina School of Medicine, Chapel Hill, North Carolina, United States of America; University of Connecticut School of Medicine, UNITED STATES OF AMERICA

## Abstract

In this study, we investigated the hemodynamic characteristics of arteriovenous fistulas (AVFs) in murine models using micro-CT based computational fluid dynamics (CFD). By combining high-resolution micro-CT imaging with ultrasound flow measurements, our methodology offers a cost-effective and efficient alternative to traditional MRI-based approaches. CFD simulations performed at 7 and 21 days post-surgery revealed substantial temporal changes in both geometry and hemodynamics. Geometric analysis showed that the proximal artery diameter increased from 0.29 mm to 0.38 mm, whereas the initial 2 mm fistula segment showed a 21.6% decrease (0.74 mm to 0.58 mm). Blood flow through the AVF nearly doubled from 1.33 mL/min to 2.57 mL/min. Time-averaged wall shear stress (TAWSS) peak values and locations changed from 142 Pa (day 7) within the proximal artery to 200 Pa (day 21) in the stenotic region. The oscillatory shear index (OSI) showed marked elevation at the anastomosis (increasing from 0.22 to 0.48), indicating disturbed flow development. An inverse relationship between TAWSS and OSI was identified, consistent with previous studies. Our methodology demonstrates the capability to analyze relationships between early hemodynamics and subsequent geometric changes. This approach can enable identification of regions susceptible to stenosis development and monitoring of AVF maturation, which can ultimately lead to quantitative metrics to evaluate surgical outcomes and early therapeutic interventions.

## Author summary

For end-stage renal disease patients who require hemodialysis, an arteriovenous fistula (AVF), which provides a direct connection between an artery and a vein, is a preferred vascular access method. Unfortunately, these connections frequently fail to mature properly, mainly due to neointimal hyperplasia smooth muscle cells, leading to narrowing of the vessel (stenosis) in the venous segment of the AVFs, along with other complications. In this study, we developed a new method to analyze blood flow patterns in AVFs. By combining high-resolution CT scans with ultrasound measurements in mice, we created detailed computational models showing how blood flows through these connections at different timepoints after surgery. We also assessed the possibility of establishing predictive relationships between early hemodynamic parameters and susceptible regions for late stenosis development. Our approach offers a more affordable and accessible alternative to traditional imaging methods and can potentially facilitate predictions regarding AVF maturation. With further development, this technology may eventually guide surgical techniques to create more successful connections, leading to improved outcomes for hemodialysis patients.

## Introduction

An arteriovenous fistula (AVF) is a direct connection between an artery and a vein that can occur naturally or be created through surgery. Surgically created AVFs are primarily used for hemodialysis access in patients with end-stage renal disease [1]. Despite being the preferred vascular access method due to superior longevity and lower complication rates compared to central venous catheters and grafts [2], AVF maturation and failure remain significant clinical challenges [3]. Maturation failure, which is defined as an inability of the AVF to increase flow and diameter adequately to support a successful clinical dialysis session, occurs in approximately 20–50% of cases, leading to increased morbidity and healthcare costs [4–6].

Understanding the hemodynamic environment within the AVF is crucial because it plays a pivotal role in both the maturation process and long-term patency [5,7], with factors such as blood flow velocity, pressure, and wall shear stress significantly influencing endothelial cell function, vascular remodeling, and the development of neointimal hyperplasia—a leading cause of AVF failure [7]. The creation of an AVF also triggers cardiac changes in murine models [8], suggesting a complex interplay between cardiac function and local hemodynamics. Previous studies suggest that stenosis and neointimal hyperplasia development are associated with low wall shear stress but high oscillatory shear index (OSI) [9,10], whereas higher wall shear stress with a lower OSI promote lumen expansion after AVF creation [11]. Despite these findings, the complete mechanistic relationships between these hemodynamic parameters and vascular remodeling are complex and have not been fully elucidated [12,13]. Further, traditional experimental methods for studying AVF hemodynamics have been limited by insufficient spatial and temporal resolution to capture complex flow dynamics [14–16].

Computational fluid dynamics (CFD) offers a powerful tool to overcome these limitations by enabling detailed simulations of blood flow within AVF [17]. By integrating anatomical data from high-resolution imaging modalities such as micro-computed tomography (micro-CT), CFD provides comprehensive insights into the hemodynamic environments within different regions of the AVF. This approach enables precise quantification of flow patterns, pressure distributions, and wall shear stress profiles, facilitating a deeper understanding of factors contributing to AVF maturation and failure [18].

Murine models serve as valuable platforms for AVF research because of their genetic similarities to humans, the availability of sophisticated genetic tools, the ability to manipulate individual genes and their downstream proteins, and cost-effectiveness [19,20]. Whereas MRI-based CFD models have been developed for murine AVF analysis [21], CT-based CFD models with ultrasound velocity data offer several distinct advantages that have not been fully explored. For geometric reconstruction, in vivo CT imaging provides both high spatial resolution and accurate vasculature morphology under live physiological or pathophysiological conditions, which are crucial for more precise computational modeling of complex AVF geometries [22,23]. Additionally, Doppler-based ultrasound methods offer significant advantages over phase-contrast MRI-based flow measurement. Although MRI-based flow measurement is usually more accurate and can provide 4D flow, Doppler ultrasound enables quick, repeated assessments with real-time feedback that allows for a detailed profile of CFD changes over time, in addition to high portability, and cost-effectiveness [24].

Our approach therefore combines the strengths of both modalities: high-resolution CT imaging for precise geometric reconstruction and Doppler ultrasound for efficient flow measurements. This combination also offers advantages over 4D flow MRI in terms of spatial resolution and reduced scan times [25,26], which is particularly beneficial for imaging small vessels near bony structures such as the neck region where AVFs are typically created.

Previous studies have characterized either the geometric or hemodynamic aspects of AVF development [16,27,28], but the relationship between early flow patterns and subsequent vascular remodeling remains poorly understood. This study aims to bridge this gap by:

(1) Developing a micro-CT-based CFD methodology for murine AVF analysis.
(2) Characterizing the temporal evolution of AVF hemodynamics with high spatial resolution.

By integrating detailed anatomical data from high-resolution imaging with advanced computational techniques, we seek to establish quantitative metrics for predicting AVF maturation outcomes. Our findings are expected to provide valuable insights into the mechanistic underpinnings of AVF development, potentially leading to future clinical interventions and improved outcomes for hemodialysis patients in the future.

## Materials and methods

### Ethics statement

All animal procedures were approved by institutional guidelines and approved by the University of North Carolina at Chapel Hill’s Institutional Animal Care and Use Committee and performed in accordance with National Institute of Health guidelines. Proper care was taken to minimize animal suffering.

### Animal model

This study used male C57BL/6J mice aged 14–16 weeks that were obtained from The Jackson laboratory. Imaging and ultrasound data were collected at days 7 and 21 post-surgery to characterize the early and late stages of AVF development.

### Surgical procedure

AVF surgery was performed following established protocols [29,30]. The mouse was anesthetized using a combination of isoflurane inhalation (1–3%) with prior surgery analgesia (buprenorphine, 0.1 mg/kg).

**Fig 1:**
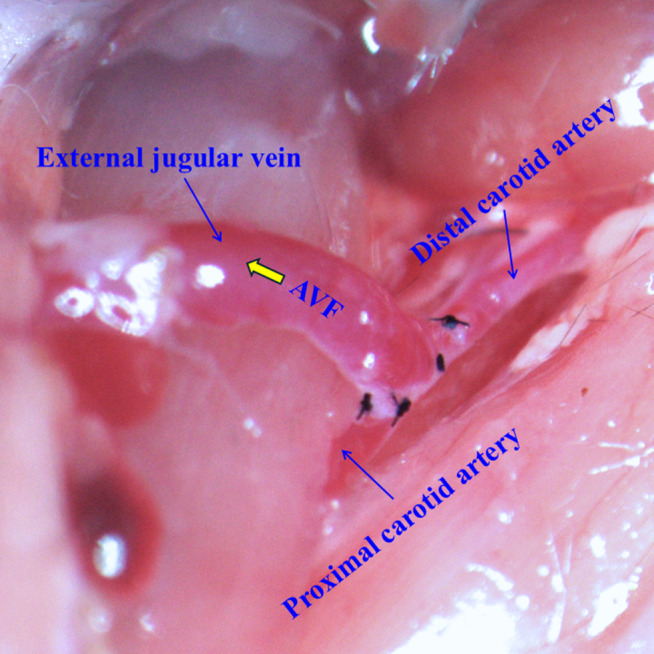
AVF creation using end-to-side anastomosis between jugular vein and carotid artery in the murine model. The white arrow shows the direction of venous outflow.

A 1 cm longitudinal incision was made in the middle of the neck. Under a surgical microscope (12.5-25X magnification), the right external jugular vein and common carotid artery were carefully exposed through blunt dissection. The jugular vein was ligated distally and divided, while the carotid artery was temporarily clamped using micro-vascular clamps. An end-to-side anastomosis was created between the jugular vein and carotid artery: first, an arteriotomy (approximately 0.4-0.5 mm) was made in the carotid artery, then the jugular vein was anastomosed to the artery using interrupted 10–0 nylon sutures. Fig 1 illustrates the surgical creation of the AVF, showing the end-to-side anastomosis between the jugular vein and carotid artery.

### Imaging protocol

High-resolution micro-computed tomography (micro-CT) imaging was performed in vivo at days 7 and 21 post-surgery using a Quantum GX2 system (Revvity; Waltham, MA USA) with the following parameters: 90 kV of peak energy, 88 μA of current, 72 μm isotropic voxel size, and 2 min of acquisition time. For day 21 imaging, an additional scan with 14 min acquisition time was performed to achieve a high signal-to-noise ratio. Prior to imaging, either Fenestra-HDVC (day 7) or mvivoAU (day 21) (MediLumine Inc, Montreal, Quebec CA) was administered intravenously via a tail vein catheter (5 µl/g for both agents) to provide vascular contrast for vessel segmentation.

### Blood flow velocity acquisition

Prior to micro-CT imaging, a high-frequency Doppler ultrasound system (Vevo F2, FUJIFILM VisualSonics) with a UHF57x linear array transducer with center frequency at 32 MHz was used to obtain blood flow velocity measurements on the same day. Mice were anesthetized using 1.5-2% isoflurane, and body temperature was maintained at 37°C during measurements.

Velocity measurements were obtained using pulsed-wave Doppler mode imaging at three locations: the proximal artery (1.5 mm upstream of anastomosis), distal artery (1.5 mm downstream of anastomosis), and venous segment of the AVF. The angle between the wave beam and flow direction was kept below 60° either by either animal positioning or by using additional 15° beam steering. At each location, pulsatile velocity waveforms were recorded over multiple cardiac cycles. Three consecutive cycles were extracted and averaged to generate representative velocity profiles for each location. Peak velocity in mm/s was determined for each location. The volumetric flow rate was calculated using Q=AV, in which A is the cross-sectional area obtained from the micro-CT data and V is the mean velocity. The cross-sectional velocity was assumed to follow a parabolic profile. This assumption is justified by the low Womersley number (≈ 1) in this study, which indicates that viscous effects dominate over inertial effects, making the parabolic profile a reasonable approximation for pulsatile flow in small vessels [31]. Heart rate was monitored throughout the measurements and remained stable at approximately 450 beats per minute. The acquired velocity data served as boundary conditions for the subsequent CFD simulations.

### Image processing and geometry reconstruction

**Fig 2:**
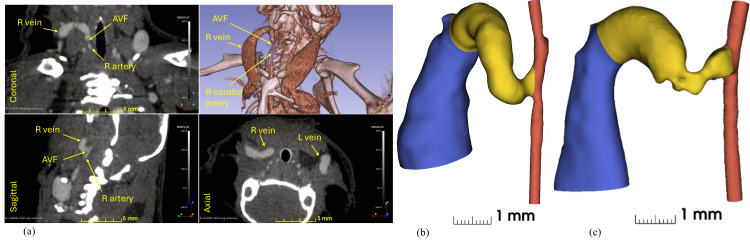
Contrast-enhanced CT images and segmented vascular structure. (a) CT images in three orientations, coronal, sagittal, and axial, near the AVF. A volume rendering of the CT images is shown in the upper right to visualize vasculature and bone structure. The AVF and surrounding veins and arteries are annotated in yellow. (b) and (c) Segmented vasculature near the AVF at day 7 and day 21 post-surgery, respectively. Color coding represents distinct vessel segments: red indicates the carotid artery (with the proximal segment at bottom and distal segment at top), yellow highlights the AVF region (4 mm of length), and blue shows the main venous drainage with its characteristic ballooning of the vein starting 4 mm from the anastomosis.

Image processing and three-dimensional vessel reconstruction were performed using a multi-step approach. The micro-CT scans were first processed in ITK-SNAP (Version 4.2) [32,33] for initial segmentation (Fig 2A).

The vascular lumen was segmented using a combination of threshold-based and region-growing techniques. Initial thresholding (300–1000 HU) isolated contrast-enhanced vessels and bones from the neck area, followed by seed-based region-growing to further segment connected voxels to form vessel lumens. Manual editing was performed where necessary, particularly at the anastomosis region to ensure accurate geometry capture.

The segmented vasculature was exported as STL files and further processed in Autodesk Meshmixer [34] to remove artifacts and smooth surface irregularities. The inlet and outlet regions of the artery were extended by 2 mm to ensure fully developed flow, and geometric landmarks were defined for consistent analysis.

Centerline analyses were performed using the VMTK module (Vascular Modeling Toolkit [35]) in 3D Slicer [36] to extract vessel centerlines and calculate cross-sectional areas and diameters. These measurements provided geometric metrics for quantitative comparison between timepoints. The final reconstructed geometry consisted of the proximal artery (inflow), distal artery (outflow), and the arteriovenous fistula, which was defined as the 4 mm length of vessel extending from the anastomosis, as shown in Fig 2B and 2C.

### Computational fluid dynamics

All simulations used OpenFOAM v2306 [37], an open-source finite volume based computational fluid dynamics software package. OpenFOAM provides numerical solvers, utilities, and tools for solving continuum mechanics problems involving fluid flow, chemical reactions, and heat transfer, among others [38]. Simulations were performed on a single node of a high-performance computing cluster with 64 cores (AMD EPYC 7313 @ 3.6 GHz) and 300 GB allocated memory. The computational cost scaled approximately linearly with the number of mesh elements and timesteps, with the medium mesh (~1.3 million elements) simulations requiring 60–72 hours of wall clock time to complete three cardiac cycles.

### Governing equations

The blood flow within the arteriovenous fistula is modeled using the incompressible Navier-Stokes equations:


$$\rho \left(\frac{\partial u}{\partial t}+u\cdot \nabla u\right)=-\nabla p+\mu \nabla^{2}u$$ (Eq 1)

∇·u=0 (Eq 2)

in which ρ is the density of blood (1,040 kg/m³), μ is the dynamic viscosity of blood ($4\times 10^{-3}Pa\cdot s$), u is the velocity vector of the blood flow, p is pressure, and t is time.

In this study, blood was modeled as a Newtonian fluid, which is an appropriate assumption because the vessel diameters (0.3-1.0 mm) exceed the threshold (0.2-0.3 mm) where non-Newtonian effects become significant [39,40]. Additionally, the wall shear rates in our regions of interest (anastomosis and stenosis) predominantly exceed 200 s ⁻ ¹, which is a shear rate at which blood is known to exhibit nearly Newtonian behavior [40]. This assumption aligns with our comparative analysis approach, focusing on relative hemodynamic patterns between timepoints rather than absolute values. The non-Newtonian behavior of blood (primarily due to red blood cell aggregation and deformation) becomes most significant in smaller vessels [41], particularly when vessel diameter approaches the scale of blood cells [42].

We employed a rigid wall assumption throughout this study, which is reasonable given that arteriovenous fistula wall displacement during the cardiac cycle is typically small compared to vessel diameter, particularly in mature fistulas with thickened walls [43].

### Mesh generation and grid independence

**Fig 3:**
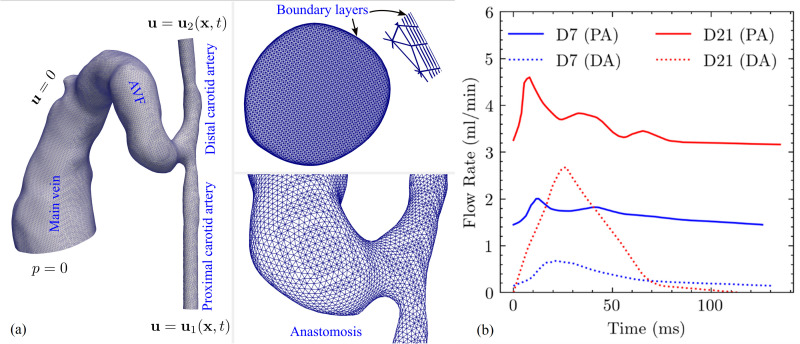
Computational model setup and flow rate boundary conditions for murine AVF analysis. (a) Computational domain, boundary conditions, and mesh configuration. The three-dimensional computational domain represents the murine carotid artery-jugular vein arteriovenous fistula model (day 7 geometry shown). Boundary conditions include: velocity-based inlet conditions at both proximal and distal carotid artery ends derived from ultrasound Doppler measurements, no-slip conditions at all vessel walls, and zero-pressure outlet conditions at the venous exit. The computational mesh consists of tetrahedral elements with five prismatic boundary layers near vessel walls (upper inset) to capture near-wall flow gradients. The lower inset shows detailed mesh refinement at the arteriovenous junction. (b) Flow rate waveforms for boundary condition implementation. Pulsatile flow rate waveforms for the proximal artery (PA) and distal artery (DA) derived from ultrasound Doppler velocity measurements at day 7 and day 21 post-fistula creation. These waveforms serve as time-varying inlet boundary conditions for the computational fluid dynamics simulations, capturing the physiological flow patterns and their temporal evolution during fistula maturation.

The computational domain encompasses the proximal artery, distal artery, fistula (4 mm length from anastomosis) (Fig 3A), and vein. The domain was discretized using tetrahedral meshes with five prismatic boundary layers (first layer thickness 1–3 µm, growth ratio 1.2) near the walls to ensure accurate flow gradient calculations.

**Fig 4:**
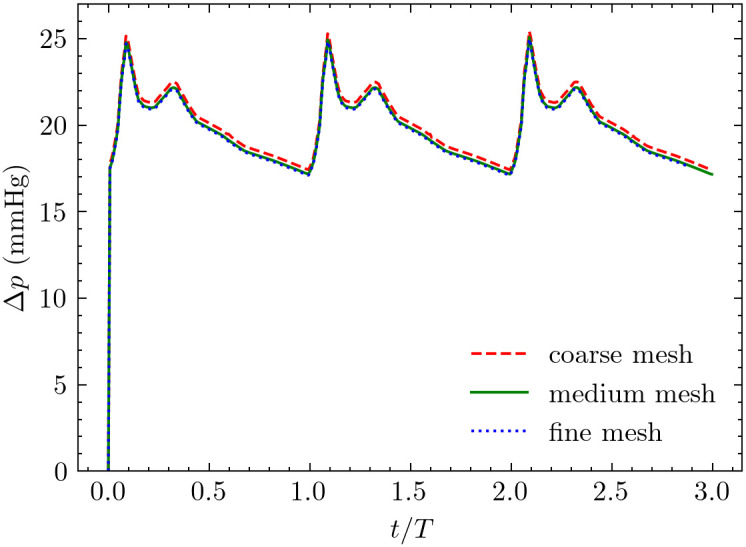
Grid convergence and temporal convergence analysis. Pressure time history at the proximal inlet demonstrates: (i) grid independence between medium and fine meshes, with differences less than 1% throughout the cardiac cycle and (ii) periodic steady-state was achieved after two cycles, with the first cycle influenced by initial conditions. Data from the third cycle, computed with medium mesh resolution, was used for all subsequent analyses.

Grid independence was established using three mesh resolutions for the day-7 model: coarse (0.62M elements, average spacing ~4 μm near walls, ~ 50 μm in bulk); medium (1.34M elements, average spacing ~2 μm near walls, ~ 30 μm in bulk); and fine (2.52M elements, average spacing ~1.5 μm near walls, ~ 20 μm in bulk). Convergence was assessed by comparing velocity magnitude and pressure distributions at multiple cross-sectional planes throughout the domain, including the inlet sections, the anastomosis, stenotic region, and downstream sections. Point-wise differences were calculated using the maximum norm between consecutive mesh refinements [44] at identical spatial locations during peak systole. The maximum point-wise differences between medium and fine meshes were below 1% for both velocity and pressure fields across all evaluation planes and timepoints, confirming grid convergence. Fig 4 illustrates the pressure comparison at the proximal inlet across three sets of mesh; other flow quantities demonstrated similar convergence behavior. Following this analysis, the medium mesh resolution was adopted for all analyses, corresponding to 1.34M and 1.97M elements for day-7 and day-21 models, respectively, with average grid spacing of approximately 2 μm near the wall and 30 μm away from the wall.

### Boundary conditions and numerical methods

Velocity boundary conditions derived from ultrasound Doppler measurements were applied at artery inlets (Fig 3B), and zero-pressure conditions imposed at the venous outlet. No-slip conditions were enforced at vessel walls. Parabolic profiles were used in the numerical model, consistent with the experimental data analysis described in *Blood flow velocity acquisition*. Additionally, our model implemented extended inlet sections exceeding 4 diameters in length, allowing the flow to fully develop before reaching the region of interest. Previous work [45] has shown that different inlet velocity profiles (including plus flow, parabolic flow, and Womersley profiles) converge within 1–2 diameters downstream in CFD models, and our analysis primarily focuses on the AVF region and anastomosis, which are positioned at least 10–15 diameters downstream from the prescribed inflow and outflow velocity profiles. This approach balances computational efficiency with accuracy in the regions of interest, which is particularly important for our comparative temporal analysis.

Although impedance-based outflow conditions such as the Windkessel model can provide more physiologically realistic representations of downstream vascular networks, we applied a zero-pressure (free outflow) condition at the venous outlet. This choice is consistent with the rigid wall assumption employed throughout this study. Under the rigid wall modeling framework with prescribed inflow velocities, whether a simple boundary condition (zero pressure) or a complex one (Windkessel model) was implemented, the choice only affected the absolute pressure values within the domain. All pressures reported herein are relative to the outlet pressure. The zero-pressure boundary condition is therefore preferable as it introduces no additional fittable parameters to the modeling pipeline while maintaining consistency with our rigid wall assumptions and comparative analysis approach focusing on relative hemodynamic patterns between timepoints.

The time discretization employed the backward Euler scheme for temporal derivatives, and the nonlinear convective terms were handled through iterative coupling within each time step. The time step size was adjustable during the simulations to ensure the advective CFL number was not greater than 0.1. The spatial discretization used second-order upwind schemes for the convective terms and Gauss linear gradient calculations for the diffusive terms. The iterative PIMPLE algorithm (a combination of PISO and SIMPLE) handles pressure-velocity coupling, with convergence achieved once the absolute residual for all governing equations (continuity and momentum) drops below 10^-6^. Simulations ran for three cardiac cycles, and periodic steady-state was achieved after two cycles, with the first cycle influenced by initial conditions; therefore, only the final cycle was used for analysis.

### Data analysis methods

#### Hemodynamic parameters.

Several hemodynamic parameters were calculated to characterize the flow field and its potential influence on vascular remodeling. The wall shear stress (WSS), representing the tangential force exerted by blood flow on the vessel wall, was computed as


$$WSS=\tau ={\;\mu \frac{\partial v}{\partial n}|}_{wall}$$ (Eq 3)

in which v is the velocity component parallel to the vessel wall and n is the unit vector normal to the wall.

The time-averaged wall shear stress (TAWSS) is calculated over one cardiac cycle (T) as


$$TAWSS=\frac{1}{T}\int_{0}^{T}|\tau |\;dt$$ (Eq 4)

TAWSS provides a measure of the chronic mechanical load experienced by endothelial cells [7]. In AVF development, regions of consistently high TAWSS often trigger outward vascular remodeling as an adaptive response, whereas regions of low TAWSS may be prone to intimal hyperplasia development [21].

The oscillatory shear index (OSI) quantifies the directional changes in wall shear stress over the cardiac cycle and is computed as


$$OSI=\frac{1}{2}\left(1-\frac{\left|\int_{0}^{T}\tau \;dt\right|}{\int_{0}^{T}|\tau |\;dt}\right)=\frac{1}{2}\left(1-\frac{\frac{1}{T}\left|\int_{0}^{T}WSS\;dt\right|}{TAWSS}\right)$$ (Eq 5)

OSI ranges from 0 to 0.5, in which 0 indicates unidirectional flow and 0.5 represents complete flow reversal. In AVF, high OSI regions often correlate with disturbed flow patterns and are frequently associated with stenosis development [46]. This is particularly significant at the anastomosis and in post-stenotic regions where complex flow patterns emerge [23].

The vortex structure is commonly discerned using the Q-criterion, computed as the second invariant of the velocity gradient tensor:


$$Q=\frac{1}{2}\left({\left\|\Omega \right\|}^{2}-{\left\|S\right\|}^{2}\right)$$ (Eq 6)

in which **Ω** and **S** are the antisymmetric and symmetric components of the velocity gradient tensor, respectively. Positive values of the Q-criterion indicate regions where rotation dominates strain, identifying coherent vortical structures [22].

### Data analysis

Cross-sectional averages and maxima of hemodynamic parameters were computed at key locations along the vessel centerline. Temporal comparisons between day 7 and day 21 were performed for both geometric and hemodynamic parameters. Correlations between early hemodynamic conditions and subsequent geometric changes were analyzed to identify potential predictive relationships.

## Results and discussion

### Clinical analysis

#### Temporal changes in vessel geometry.

Table 1 summarizes the lumen diameters of the carotid artery, fistula, and vein. For each segment, the values in parentheses indicate the minimum and maximum diameters. Additionally, detailed statistical analyses of the AVF region are provided for two specific segments: the initial 2 mm segment adjacent to the anastomosis and the extended 4 mm segment, to characterize the spatial variation in remodeling patterns.

**Table 1:** Diameters of the AVF models for day 7 and day 21 (unit: mm).

Day	Proximal Artery	Distal Artery	AVF (2 mm)	AVF (4 mm)
**7**	0.29 (0.23 - 0.35)	0.36 (0.27 - 0.51)	0.74 (0.46 - 0.95)	0.88 (0.46 - 1.07)
**21**	0.39 (0.34 - 0.54)	0.46 (0.40 - 0.52)	0.58 (0.31 - 0.96)	0.86 (0.31 - 1.71)

Temporal analysis revealed distinct changes in vessel geometry between day 7 and day 21. The proximal carotid artery showed a uniform expansion, with the average diameter increasing from 0.29 mm to 0.38 mm. In contrast, the fistula demonstrated more complex remodeling patterns that varied by region. Analysis of different fistula segments revealed varying degrees of remodeling: although the average diameter over the initial 4 mm segment remained relatively stable (slight decrease from 0.88 mm to 0.86 mm), a more focused analysis of the initial 2 mm segment showed notable narrowing, with the average diameter decreasing by 21.6% from 0.74 mm to 0.58 mm. The most severe narrowing was observed at the point of minimum diameter, which decreased substantially from 0.46 mm to 0.31 mm (32.6%). The vein exhibited the most dramatic change in the opposite direction, with its maximum diameter expanding from 1.07 mm to 1.71 mm (59.8%).

**Fig 5:**
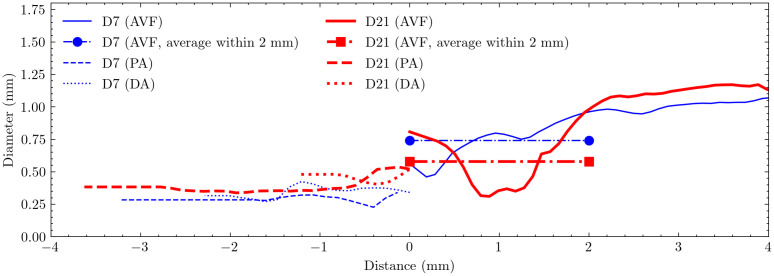
Lumen diameter profiles along the vascular segments at day 7 (D7) and day 21 (D21). The x-axis represents the distance from the anastomosis, with negative values indicating upstream segments (proximal artery [PA] and distal artery [DA]) and positive values indicating downstream segments (arteriovenous fistula [AVF]). The average AVF diameter is measured within the first 2 mm of the fistula. Cross-sections F1-F4, spaced 0.5 mm apart, indicate locations analyzed for hemodynamics in results.

Fig 5 compares the longitudinal profiles of lumen diameter on day 7 and day 21. Both timepoints show a characteristic narrowing downstream of the anastomosis, but with distinct patterns. On day 7, the narrow region was localized and positioned close to the anastomosis. By day 21, the vessel geometry had remodeled such that the immediate anastomosis region showed expansion, while the stenotic segment had both elongated and shifted further downstream. This temporal evolution of the stenosis pattern suggests dynamic vascular remodeling during the maturation process, which has been also observed in human AVFs [47].

### Flow rates

Flow analysis revealed a substantial temporal increase in total flow, with the AVF flow rate nearly doubling from 1.33 mL/min at day 7 to 2.57 mL/min at day 21, indicating fistula maturation. The flow distribution pattern demonstrated that the AVF served as the primary conduit at both timepoints, with 80% of proximal artery flow directed through the AVF at day 7, decreasing slightly to 70% by day 21, suggesting stenosis development within the AVF. Consequently, the proportion of blood flow through the distal artery increased from 20% to 30% between day 7 and day 21. Note that the blood in the distal artery flowed away from, rather than toward, the AVF junction. The proximal artery flow rate doubled over this period, while the heart rate remained at approximately 450 beats per minute throughout the study period.

The typical carotid artery flow rate in normal mice ranges from 0.3 to 2.1 mL/min [48]. A 10–20-fold increase in blood flow rate is considered necessary for sufficient AVF maturation in humans [49]. Therefore, the flow rate increase observed in this study suggests that the AVF may not have achieved full maturation, possibly due to the development of stenosis.

### Hemodynamics analysis

#### Flow field characteristics.

**Fig 6:**
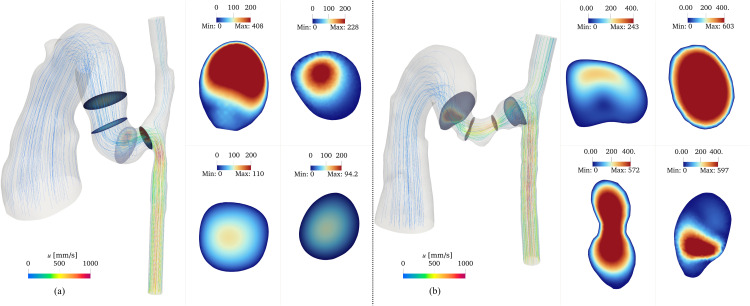
Flow patterns within the AVF at peak systole for day 7 (a) and day 21 (b) models. Streamlines colored by velocity magnitude show the three-dimensional flow trajectories. Cross-sectional velocity distributions at representative locations demonstrate the spatial evolution of the flow field. The color scale represents velocity magnitude in mm/s. Cross-sectional dimensions are not to scale.

Flow field analysis revealed distinctive patterns at both timepoints that are characterized by complex three-dimensional structures. Fig 6 uses streamlines to illustrate flow patterns and velocity distributions at peak systole. Flow separation occurred at regions of high curvature and abrupt lumen changes, resulting in asymmetric velocity profiles at these locations.

Comparing velocity fields between the two timepoints and across different anatomical locations revealed distinct velocity patterns. At the anastomosis, the flow velocity was higher on day 7 (maximum velocity 408 mm/s) compared to day 21 (maximum velocity 243 mm/s), with pronounced asymmetry between the upper and lower regions. In contrast, at the stenotic region, day 21 exhibited substantially higher velocities (maximum 603 mm/s) compared to day 7 (maximum 228 mm/s), consistent with the increased flow constriction.

The flow symmetry showed different recovery patterns between the two timepoints. On day 7, the flow velocity profile became symmetric beyond the 2 mm fistula region. However, on day 21, flow asymmetry persisted beyond this region, as evidenced by both streamline patterns and cross-sectional velocity distributions. Further downstream, in the main vein, the flow exhibited axisymmetric patterns in cross-sectional views, with peak velocities concentrated along the vessel centerline, consistent with developed flow characteristics in straight vessel segments.

**Fig 7:**
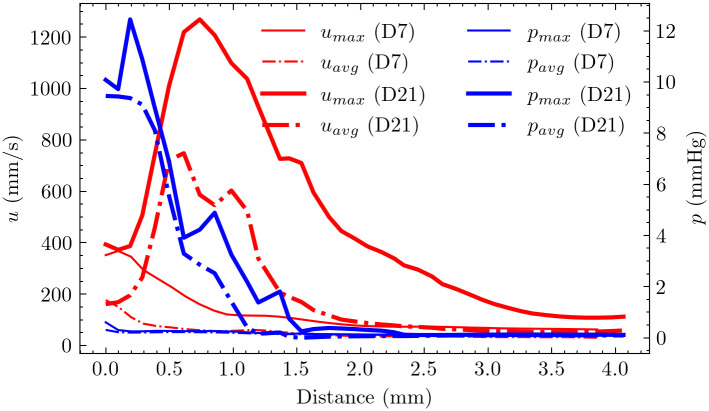
Cross-sectional average velocity and pressure along the vessel centerline at peak systole for day 7 and day 21 models. The plots show velocity (red) and pressure (blue) distributions relative to distance from the anastomosis. Both models exhibit characteristic features: a sharp velocity increase preceding the stenosis followed by deceleration, with large velocities occurring 1 mm downstream of the anastomosis. A corresponding abrupt pressure drop is observed immediately before the stenotic region. The pressure drop completes (99%) within 1.5 mm downstream of the anastomosis on day 21, whereas it only occurs 50% over the same distance on day 7.

Fig 7 presents the centerline analysis of velocity and pressure, demonstrating characteristic flow features: velocity sharply increased before the stenosis, followed by deceleration, with peak velocities occurring within 1 mm downstream of the anastomosis. Consistent with the Bernoulli principle, an abrupt pressure drop was observed immediately preceding the stenotic region. Notably, pressure drop characteristics differed between timepoints: at day 21, 99% of the total pressure drop occurred within 1.5 mm downstream of the anastomosis, compared to only 50% at day 7 over the same distance.

According to Poiseuille’s Law, the calculated flow resistance in the AVF segment (4mm length) on day 21 was approximately 9 times greater than that on day 7. This substantial increase in flow resistance indicates substantially higher friction forces, indicative of the more severe stenosis developed by day 21.

### Wall shear stress analysis

**Fig 8:**
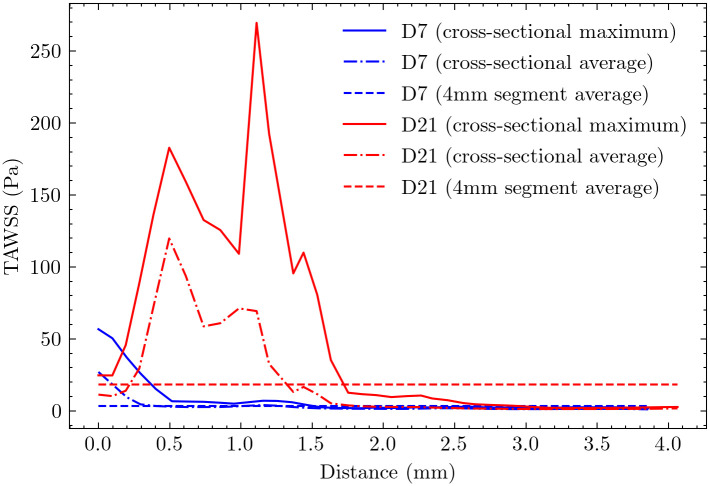
Cross-sectional maximum and average distributions of TAWSS along the centerline for day 7 and day 21. The two horizontal lines represent the average values across the first 4 mm AVF. For day 7, peak TAWSS is localized within 0.5 mm downstream from the anastomosis, with an average value of 3 Pa across the 4 mm fistula region. By day 21, peak TAWSS shifts to 1.2 mm downstream from the anastomosis, with the average value increasing sixfold to 18 Pa across the 4 mm fistula region.

Fig 8 illustrates the temporal and spatial evolution of TAWSS along the AVF centerline. On day 7, TAWSS distribution shows distinct spatial patterns, reaching its peak within 0.5 mm downstream from the anastomosis, with the 4 mm fistula region showing an average value of 3 Pa. This localized peak suggests early flow adaptation near the surgical connection.

By day 21, TAWSS showed significant changes in both magnitude and distribution. The peak TAWSS location shifted further downstream to 1.2 mm from the anastomosis, and its average value increased substantially to 18 Pa—a sixfold increase from day 7. This shift in peak location and magnitude suggests progressive vascular remodeling and potential stenosis development.

**Fig 9:**
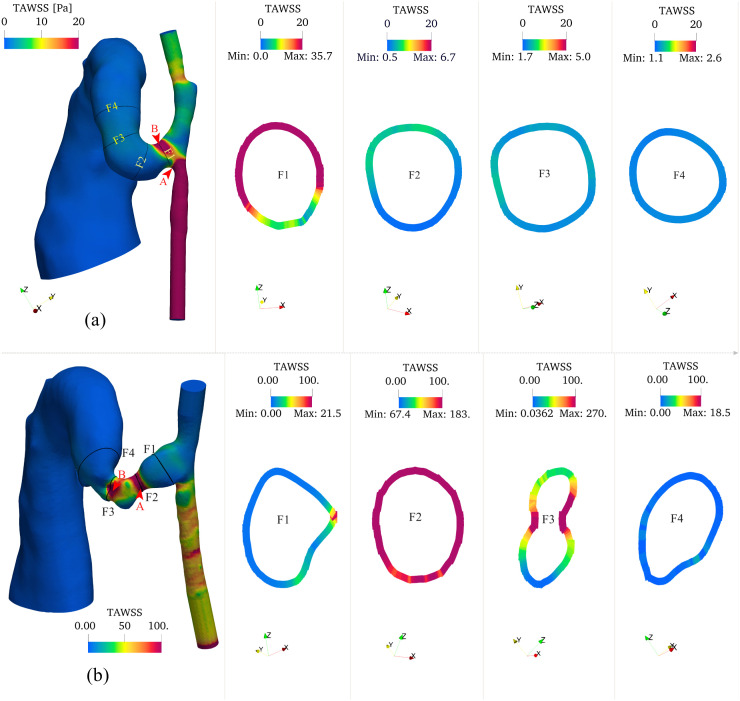
Time-averaged wall shear stress (TAWSS) distributions of (a) day 7 and (b) day 21 models. Each panel shows three-dimensional TAWSS colormaps along the vessel surfaces and representative cross-sectional (F1 to F4) distributions showing circumferential variation of TAWSS at key locations (anastomosis, stenosis, and AVF). TAWSS exhibits pronounced spatial heterogeneity, with peak values localized at the stenotic region. A significant temporal increase in TAWSS magnitude is observed from day 7 to day 21, particularly in regions of geometric constriction. The color scale represents TAWSS in Pa. Points A and B (indicated by arrows) show locations of maximum TAWSS values: 142 Pa and 37 Pa on day 7, and 134 Pa and 200 Pa on day 21, respectively. Cross-sectional dimensions are not to scale.

The three-dimensional TAWSS distribution (Fig 9) revealed further spatial variations. On day 7, peak TAWSS values were observed on the artery wall, with the maximum occurring at the inner side of the anastomosis on the proximal artery wall (Point A, 142 Pa) and a secondary peak at the stenosis (panel (a) Point B, 37 Pa). The proximal and distal arteries showed average TAWSS values of approximately 50 Pa and 4 Pa, respectively. By day 21, the TAWSS distribution pattern shifted substantially due to severe stenosis development in the fistula. The maximum TAWSS relocated to the AVF wall (panel (b) Point B, 200 Pa), with a secondary peak at the first stenosis (Point A, 134 Pa). The proximal and distal artery TAWSS values increased moderately to 60 Pa and 5 Pa, respectively.

### Oscillatory shear index analysis

**Fig 10:**
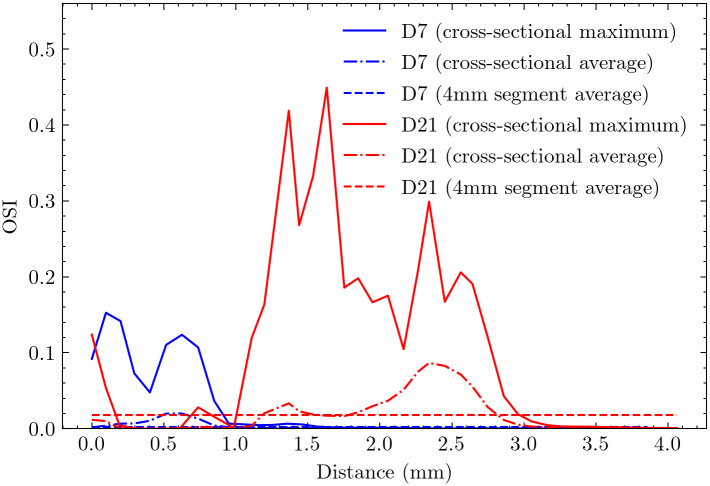
Cross-sectional maximum and average distributions of OSI along the centerline for (a) day 7 and (b) day 21. The two horizontal lines represent the average values across the first 4mm AVF. For day 7, peak OSI occurs within 1.0 mm downstream from the anastomosis, with an average value of 0.0025 across the 4 mm fistula region. By day 21, peak OSI extends from 1.0 to 2.5 mm downstream from the anastomosis, with the average value increasing substantially to 0.016 across the 4 mm fistula region.

The centerline analysis of OSI (Fig 10) reveals distinct temporal evolution patterns. On day 7, the maximum OSI occurred within 1.0 mm downstream from the anastomosis, with a relatively low average value of 0.0025 across the 4 mm fistula region, indicating minimal flow oscillation at the early stage.

By day 21, the OSI distribution showed extensive changes, with elevated values spanning a broader region from 1.0 to 2.5 mm downstream from the anastomosis. The average OSI increases more than six-fold to 0.018 across the 4 mm fistula region, indicating the development of more complex, disturbed flow patterns.

**Fig 11:**
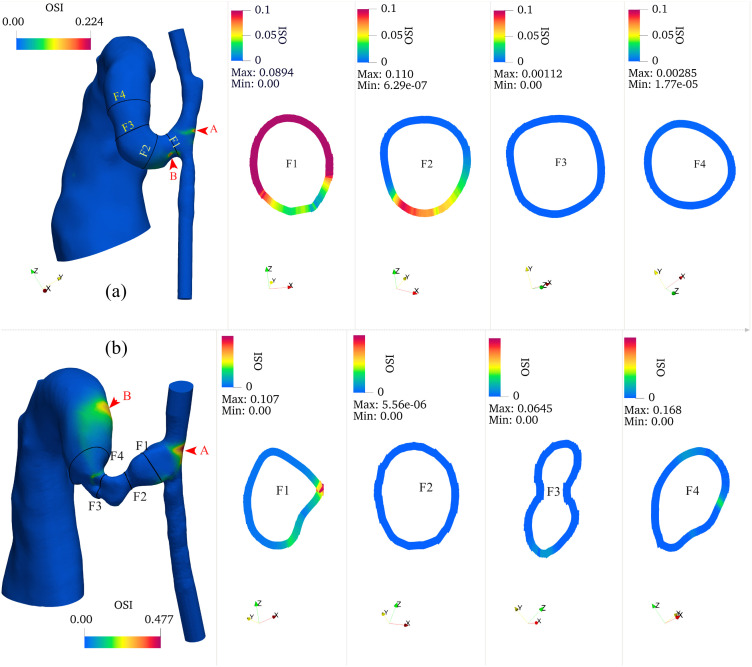
Oscillatory Shear Index (OSI) distributions of (a) day 7 and (b) day 21 models. Each panel shows three-dimensional OSI colormaps along the vessel surfaces and representative cross-sectional distributions showing circumferential variation of OSI at key locations (anastomosis, stenosis, and AVF). High OSI regions are predominantly localized at the anastomosis and downstream of the stenosis. OSI demonstrates circumferential heterogeneity, with elevated values corresponding to regions of flow separation. An obvious temporal increase in OSI magnitude is observed from day 7 to day 21. The color scale represents dimensionless OSI values from 0 to 0.1. Points A and B (indicated by arrows) show locations of maximum OSI values: 0.22 and 0.17 on day 7, increasing to 0.48 and 0.26 on day 21, respectively. Cross-sectional dimensions are not to scale.

The three-dimensional OSI distribution (Fig 11) showed that OSI remained consistently low along the artery wall except near the anastomosis region. High OSI regions were primarily concentrated in two areas: at the anastomosis and downstream of stenotic segments, showing an inverse spatial relationship with TAWSS. The circumferential OSI distribution showed marked heterogeneity, particularly in regions of flow separation, indicating asymmetric flow behavior. Temporal analysis revealed a substantial increase in OSI magnitude from day 7 to day 21, most notably at the outside of the anastomosis (Point A, increasing from 0.22 to 0.48) and at Point B (increasing from 0.17 to 0.26).

### Vortical structure

**Fig 12:**
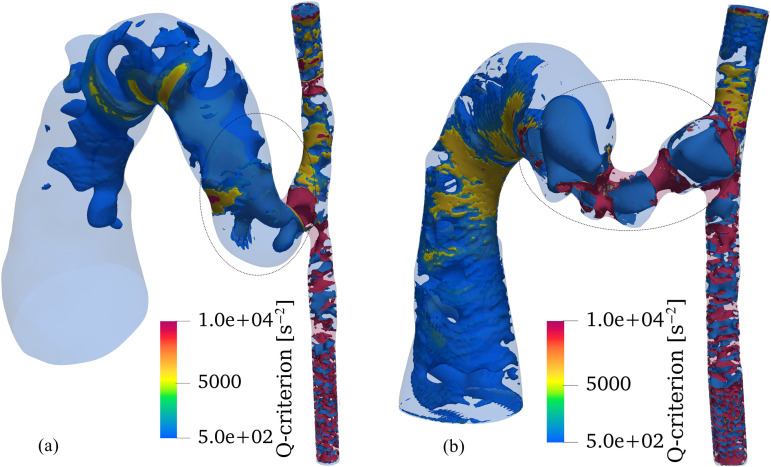
Vortical structures visualized using the Q-criterion for (a) day 7 and (b) day 21 models. Whereas vortical structures predominantly develop near vessel walls, a distinct vortex core (marked by circle) forms within the AVF vein lumen. Temporal comparison reveals increased vortical intensity on day 21 compared to day 7, particularly pronounced in stenotic regions. Although relatively small in magnitude, vortical structures are also observed to develop within the main vein. The color scale represents Q-criterion magnitude ($s^{-2}$), with positive values indicating regions where rotation dominates strain.

The development and evolution of vortical structures were analyzed using the Q-criterion (Fig 12). At both timepoints, vortical structures concentrated predominantly near vessel walls, especially in regions of geometric transition, such as the anastomosis and stenotic segments. These wall-adjacent vortices developed primarily because of flow separation and adverse pressure gradients associated with the complex geometry. Notably, a distinct vortex core formed within the AVF vein lumen, indicating substantial secondary flow patterns.

The intensity and spatial distribution of vortical structures showed marked changes by day 21. The most noteworthy intensification occurred in stenotic regions, where the combination of flow acceleration and deceleration generated stronger rotational flow patterns. This enhanced vortical activity suggests increased flow complexity and higher energy dissipation in these regions. Although vortical structures were also present in the main vein, their magnitude remained modest compared to those in stenotic regions and near-wall areas.

### Correlation analysis

**Fig 13:**
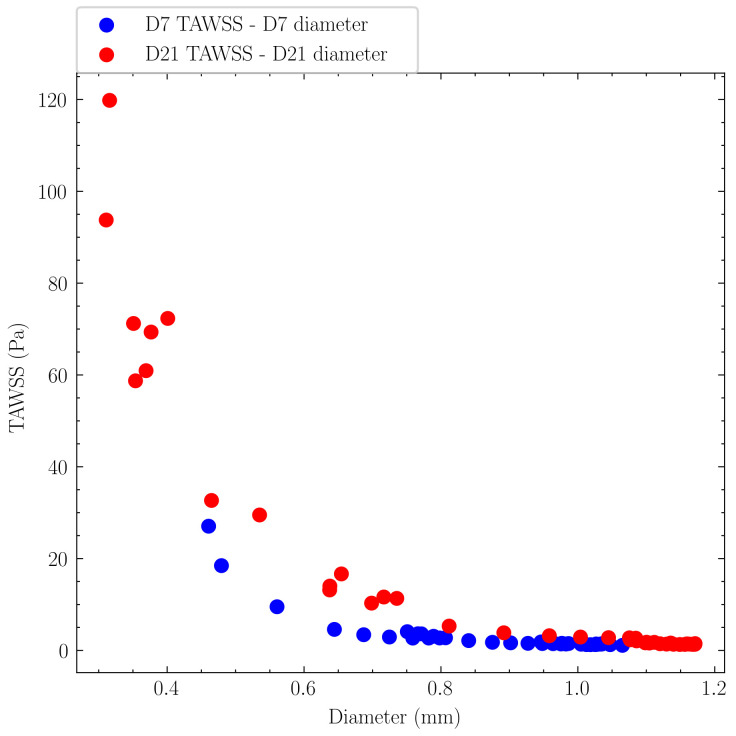
Same-day correlations between TAWSS and AVF lumen diameter. A strong nonlinear inverse relationship with diameter on both days was observed.

The relationship between vessel geometry and hemodynamic parameters was analyzed through same-day correlations. Same-day correlations (Fig 13) showed a strong inverse relationship between TAWSS and vessel diameter, following fluid dynamic principles whereby smaller diameters exhibited higher wall shear stress under constant flow.

An inverse relationship between TAWSS and OSI was observed (not shown), consistent with previous findings in various vascular studies [46,50], supporting the validity of our model.

Although the limited sample size prevented comprehensive statistical analysis of correlations between hemodynamic parameters and geometric changes, this study demonstrates the technical feasibility of identifying early hemodynamic conditions that predict subsequent remodeling and stenosis development.

The methodology established here can be applied to larger cohorts to identify associations between early hemodynamic parameters (TAWSS and OSI) and subsequent diameter changes in specific regions. This approach enables analysis of maximum and minimum stenosis at later stages (day 21) in relation to the TAWSS and OSI values at the same locations on day 7, potentially providing predictive markers for AVF maturation outcomes.

### Limitations and future directions

Several limitations of the present study should be acknowledged. First, our CFD model employed a simplified approach that assumed rigid vessel walls. Although this assumption enables efficient computational analyses, it does not fully capture vessel wall dynamics, including pulsatile deformations and wall vibrations that occur in vivo. Second, although our study demonstrates the feasibility of using CFD models to analyze hemodynamic correlations in AVF development, the small sample size limits the statistical power of our findings.

Future studies should address these limitations through two key improvements. Fluid-structure interaction (FSI) simulations will better capture the dynamic interaction between blood flow and vessel wall mechanics. Future studies incorporating FSI would necessitate determining wall material properties, parameterizing more realistic outflow boundary conditions, and significantly increasing computational resources for the simulations. While such FSI modeling would provide enhanced physiological realism, the additional complexity requires careful parameter determination for both wall mechanics and outflow impedance models. Additionally, an increased sample size will enable statistical validation of the observed correlations and potentially reveal new relationships between hemodynamic parameters and vascular remodeling, particularly in using early AVF CFD parameters to predict the progression of stenosis development and AVF maturation.

## Conclusion

This study demonstrates the utility of micro-CT-based computational fluid dynamics in characterizing the hemodynamic environment of murine AVF. Our analysis revealed substantial temporal evolution in both geometric and hemodynamic parameters from day 7 to day 21 post-surgery. The AVF flow rate nearly doubled, while the fistula exhibited complex remodeling patterns with regional variations in diameter changes. The wall shear stress analysis showed a shift in peak TAWSS locations and magnitude, accompanied by increased OSI, particularly at the region of anastomosis.

The methodology developed in this study enables analysis of relationships between early hemodynamic conditions and subsequent geometric changes, establishing a framework for identifying potential predictive indicators of stenosis development. Although our findings are derived from a mouse model with inherent hemodynamic differences from human blood flows—including lower Reynolds numbers, altered fluid-structure interactions, and different pulsatile flow characteristics—the primary contribution lies in the validated computational methodology that can be adapted for human-specific investigations. These insights into the mechanistic relationship between hemodynamics and vascular remodeling provide a foundation for future human studies that may inform strategies to improve AVF outcomes in clinical practice, potentially through optimizing anastomotic angle and geometry to achieve favorable flow profiles for enhanced AVF maturation.

Through the combination of high-resolution micro-CT imaging with ultrasound flow data, our approach not only provides a framework for future investigations into vascular adaptation and dysfunction in AVF development but also carries the potential for an easy and affordable predictive test that can inform the type (angle and curvature) of created AVF in the setting of a particular baseline artery-vein configuration.

## References

[1] Manov JJ, Mohan PP, Vazquez-Padron R. Arteriovenous fistulas for hemodialysis: Brief review and current problems. J Vasc Access. 2022;23(5):839–46. doi: 10.1177/11297298211007720 33818180

[2] Miller LM, Clark E, Dipchand C, Hiremath S, Kappel J, Kiaii M, et al. Hemodialysis tunneled catheter-related infections. Can J Kidney Health Dis. 2016;3:20254358116669129. doi: 10.1177/2054358116669129 28270921 PMC5332080

[3] Lok CE, Huber TS, Orchanian-Cheff A, Rajan DKJJ. Arteriovenous access for hemodialysis: a review. JAMA. 2024;331(15):1307-17. doi: 10.1001/jama.2024.0535 38497953

[4] Meng L, Ng JJ, Choong AMTL, Dharmaraj RB, Menon R, Wong JCL, et al. Effectiveness of a native vein arteriovenous fistula tracking system. Seminars in Dialysis. 2024;37(2):161-71. 10.1111/sdi.13179 37748774

[5] Al-Jaishi AA, Oliver MJ, Thomas SM, Lok CE, Zhang JC, Garg AX, et al. Patency rates of the arteriovenous fistula for hemodialysis: a systematic review and meta-analysis. Am J Kidney Dis. 2014;63(3):464–78. doi: 10.1053/j.ajkd.2013.08.023 24183112

[6] Turmel-Rodrigues LA, Bourquelot P, Pengloan J. Hemodialysis arteriovenous fistula maturity: US evaluation. Radiology. 2003;227(3):906–7; author reply 907. doi: 10.1148/radiol.2273021730 12773692

[7] Szafron JM, Heng EE, Boyd J, Humphrey JD, Marsden AL. Hemodynamics and Wall Mechanics of Vascular Graft Failure. Arterioscler Thromb Vasc Biol. 2024;44(5):1065–85. doi: 10.1161/ATVBAHA.123.318239 38572650 PMC11043008

[8] Ingle K, Pham L, Lee V, Guo L, Isayeva-Waldrop T, Somarathna M, et al. Cardiac changes following arteriovenous fistula creation in a mouse model. J Vasc Access. 2023;24(1):124–32. doi: 10.1177/11297298211026083 34144670 PMC9013201

[9] Browne LD, Bashar K, Griffin P, Kavanagh EG, Walsh SR, Walsh MT. The Role of Shear Stress in Arteriovenous Fistula Maturation and Failure: A Systematic Review. PLoS One. 2015;10(12):e0145795. doi: 10.1371/journal.pone.0145795 26716840 PMC4696682

[10] Ene-Iordache B, Remuzzi A. Disturbed flow in radial-cephalic arteriovenous fistulae for haemodialysis: low and oscillating shear stress locates the sites of stenosis. Nephrol Dialys Transplant. 2012;27(1):358–68. doi: 10.1093/ndt/gfr34 21771751

[11] He Y, Shiu Y-T, Imrey PB, Radeva MK, Beck GJ, Gassman JJ, et al. Association of Shear Stress with Subsequent Lumen Remodeling in Hemodialysis Arteriovenous Fistulas. Clin J Am Soc Nephrol. 2023;18(1):72–83. doi: 10.2215/CJN.04630422 36446600 PMC10101625

[12] Ding J, Du Y, Zhao R, Yang Q, Zhu L, Tong Y, et al. Detection of Abnormal Wall Shear Stress and Oscillatory Shear Index via Ultrasound Vector Flow Imaging as Possible Indicators for Arteriovenous Fistula Stenosis in Hemodialysis. Ultrasound Med Biol. 2023;49(8):1830–6. doi: 10.1016/j.ultrasmedbio.2023.04.007 37270353

[13] Hammes M, Cassel K, Boghosian M, Watson S, Funaki B, Coe F. A cohort study showing correspondence of low wall shear stress and cephalic arch stenosis in brachiocephalic arteriovenous fistula access. J Vasc Access. 2021;22(3):380–7. doi: 10.1177/1129729820942048 32693668

[14] Iftikhar M, Othman MHD, Khan IU, Ismail NJ, Imtiaz A, Mansur S, et al. Future research perspectives in hemodialysis membrane technology. J Indust Eng Chem. 2024;138:72-103. doi: 10.1016/j.jiec.2024.04.024

[15] White NA, van der Kroft SL, van der Bogt KE, Vrielink TJO, Camenzuli C, Calleja-Agius J, et al. An implantable magnetic drive mechanism for non-invasive arteriovenous conduit blood flow control. IEEE Transact Biomed Eng. 2024;71(8):2379-90. doi: 10.1109/TBME.2024.3370263 38412078

[16] Sivanesan S, How TV, Black RA, Bakran A. Flow patterns in the radiocephalic arteriovenous fistula: an in vitro study. J Biomech. 1999;32(9):915–25. doi: 10.1016/s0021-9290(99)00088-3 10460128

[17] Bennati L, Crispino A, Vergara C. Computational modeling of cardiac hemodynamics including chordae tendineae, papillaries, and valves dynamics. Comput Biol Med. 2025;186:109658. doi: 10.1016/j.compbiomed.2025.109658 39864334

[18] Hyde-Linaker G. Barrientos PH, Stoumpos S, Kingsmore DB, Kazakidi A. Patient-specific computational haemodynamics associated with the surgical creation of an arteriovenous fistula. Med Eng Phys. 2022;105:103814. doi: 10.1016/j.medengphy.2022.103814 35781379

[19] Brahmandam A, Alves R, Liu H, Gonzalez L, Aoyagi Y, Ohashi Y, et al. A central arteriovenous fistula reduces systemic hypertension in a mouse model. JVS Vasc Sci. 2024;5:100191. doi: 10.1016/j.jvssci.2024.100191 38510938 PMC10951512

[20] Liu J, Zhang D, Brahmandam A, Matsubara Y, Gao M, Tian J, et al. Bioinformatics identifies predictors of arteriovenous fistula maturation. J Vasc Access. 2024;25(1):172–86. doi: 10.1177/11297298221102298 35686495 PMC9734286

[21] Pike D, Shiu Y-T, Somarathna M, Guo L, Isayeva T, Totenhagen J, et al. High resolution hemodynamic profiling of murine arteriovenous fistula using magnetic resonance imaging and computational fluid dynamics. Theor Biol Med Model. 2017;14(1):5. doi: 10.1186/s12976-017-0053-x 28320412 PMC5360029

[22] Bozzetto M, Soliveri L, Poloni S, Brambilla P, Curtò D, Condemi GC, et al. Arteriovenous fistula creation with VasQTM device: A feasibility study to reveal hemodynamic implications. J Vasc Access. 2024;25(1):60–70. doi: 10.1177/11297298221087160 35451351 PMC10845834

[23] Niemann AK, Thrysoe S, Nygaard JV, Hasenkam JM, Petersen SE. Computational fluid dynamics simulation of a-v fistulas: from MRI and ultrasound scans to numeric evaluation of hemodynamics. J Vasc Access. 2012;13(1):36–44. doi: 10.5301/JVA.2011.8440 21725950

[24] Du Y, Ding H, He L, Yiu BY, Deng L, Yu AC, et al. Quantitative blood flow measurements in the common carotid artery: a comparative study of high-frame-rate ultrasound vector flow imaging, pulsed wave Doppler, and phase contrast magnetic resonance imaging. Diagnostics. 2022;12(3):690. doi: 10.3390/diagnostics12030690 35328242 PMC8947594

[25] Schoepf UJ, Varga-Szemes A. 4D flow meets CT: can it compete with 4D flow MRI? Radiology. 2018;289(1):59–60. doi: 10.1148/radiol.2018181210 29944084

[26] Goubergrits L, Vellguth K, Obermeier L, Schlief A, Tautz L, Bruening J, et al. CT-Based Analysis of Left Ventricular Hemodynamics Using Statistical Shape Modeling and Computational Fluid Dynamics. Front Cardiovasc Med. 2022;9:901902. doi: 10.3389/fcvm.2022.901902 35865389 PMC9294248

[27] Robbin ML, Greene T, Cheung AK, Allon M, Berceli SA, Kaufman JS, et al. Arteriovenous Fistula Development in the First 6 Weeks after Creation. Radiology. 2016;279(2):620–9. doi: 10.1148/radiol.2015150385 26694050 PMC4851120

[28] Shemesh D, Goldin I, Berelowitz D, Zaghal I, Zigelman C, Olsha O. Blood flow volume changes in the maturing arteriovenous access for hemodialysis. Ultrasound Med Biol. 2007;33(5):727–33. doi: 10.1016/j.ultrasmedbio.2006.11.019 17383804

[29] Yamamoto K, Li X, Shu C, Miyata T, Dardik A. Technical aspects of the mouse aortocaval fistula. J Vis Exp. 2013;(77):e50449. doi: 10.3791/50449 23892387 PMC3732098

[30] Yamamoto K, Protack CD, Tsuneki M, Hall MR, Wong DJ, Lu DY, et al. The mouse aortocaval fistula recapitulates human arteriovenous fistula maturation. Am J Physiol Heart Circ Physiol. 2013;305(12):H1718-25. doi: 10.1152/ajpheart.00590.2013 24097429 PMC3882542

[31] Loudon C, Tordesillas A. The use of the dimensionless Womersley number to characterize the unsteady nature of internal flow. J Theor Biol. 1998;191(1):63–78. doi: 10.1006/jtbi.1997.0564 9593657

[32] Yushkevich PA, Piven J, Hazlett HC, Smith RG, Ho S, Gee JC, et al. User-guided 3D active contour segmentation of anatomical structures: significantly improved efficiency and reliability. Neuroimage. 2006;31(3):1116–28. doi: 10.1016/j.neuroimage.2006.01.015 16545965

[33] ITK-SNAP webpage [cited 2024 5/1]. 4.2. Available from: http://www.itksnap.org/pmwiki/pmwiki.php

[34] Autodesk Meshmixer (RRID:SCR_015736). 2024 [cited 2024 6/30]. Available from: http://www.meshmixer.com

[35] The Vascular Modeling Toolkit website. 2024 [cited 2024 6/30]. Available from: http://www.vmtk.org/

[36] 3D Slicer website. 2024 [cited 2024 6/30]. Available from: https://www.slicer.org/

[37] OpenFOAM User Guide v2306. 2024 [cited 2024 6/30]. Available from: https://www.openfoam.com/

[38] Weller HG, Tabor G, Jasak H, Fureby C. A tensorial approach to computational continuum mechanics using object-oriented techniques. Comput Phys. 1998;12(6):620–31. 10.1063/1.168744

[39] Decorato I, Kharboutly Z, Vassallo T, Penrose J, Legallais C, Salsac A-V. Numerical simulation of the fluid structure interactions in a compliant patient-specific arteriovenous fistula. Int J Numer Method Biomed Eng. 2014;30(2):143–59. doi: 10.1002/cnm.2595 24493402

[40] Bernabeu MO, Nash RW, Groen D, Carver HB, Hetherington J, Krüger T, et al. Impact of blood rheology on wall shear stress in a model of the middle cerebral artery. Interface Focus. 2013;3(2):20120094. doi: 10.1098/rsfs.2012.0094 24427534 PMC3638489

[41] Sriram K, Intaglietta M, Tartakovsky DM. Non-Newtonian flow of blood in arterioles: consequences for wall shear stress measurements. Microcirculation. 2014;21(7):628–39. doi: 10.1111/micc.12141 24703006 PMC4185264

[42] Murata T. Theory of non-Newtonian viscosity of red blood cell suspension: effect of red cell deformation. Biorheology. 1983;20(5):471–83. doi: 10.3233/bir-1983-20505 6677273

[43] McGah PM, Leotta DF, Beach KW, Aliseda A. Effects of wall distensibility in hemodynamic simulations of an arteriovenous fistula. Biomech Model Mechanobiol. 2014;13(3):679–95. doi: 10.1007/s10237-013-0527-7 24037281 PMC3955413

[44] Roache PJ. Verification of codes and calculations. AIAA Journal. 1998;36:696–702. doi: 10.2514/3.13882

[45] Madhavan S, Kemmerling EMC. The effect of inlet and outlet boundary conditions in image-based CFD modeling of aortic flow. Biomed Eng Online. 2018;17(1):66. doi: 10.1186/s12938-018-0497-1 29843730 PMC5975715

[46] Moerman AM, Korteland S, Dilba K, van Gaalen K, Poot DHJ, van Der Lugt A, et al. The Correlation Between Wall Shear Stress and Plaque Composition in Advanced Human Carotid Atherosclerosis. Front Bioeng Biotechnol. 2022;9:828577. doi: 10.3389/fbioe.2021.828577 35155418 PMC8831262

[47] Li Y, He Y, Falzon I, Fairbourn B, Tingey S, Imrey PB, et al. Dynamic remodeling of human arteriovenous fistula wall obtained from magnetic resonance imaging during the first 6 months after creation. Kidn Int Rep. 2022;7(8):1905–9. 10.1016/j.ekir.2022.05.016 35967120 PMC9366358

[48] Kenwright DA, Thomson AJW, Hadoke PWF, Anderson T, Moran CM, Gray GA, et al. A Protocol for Improved Measurement of Arterial Flow Rate in Preclinical Ultrasound. Ultrasound Int Open. 2015;1(2):E46-52. doi: 10.1055/s-0035-1564268 27689153 PMC5023209

[49] Sier VQ, de Jong A, Quax PHA, de Vries MR. Visualization of Murine Vascular Remodeling and Blood Flow Dynamics by Ultra-High-Frequency Ultrasound Imaging. Int J Mol Sci. 2022;23(21):13298. doi: 10.3390/ijms232113298 36362087 PMC9655559

[50] Weinberg PD. Haemodynamic wall shear stress, endothelial permeability and atherosclerosis—A triad of controversy. Front Bioeng Biotechnol. 2022;10:836680. https://doi/10.3389/fbioe.2022.836680 35340842 10.3389/fbioe.2022.836680 PMC8948426

